# Fiber Laser Welding of Fuel Cladding and End Plug Made of La_2_O_3_ Dispersion-Strengthened Molybdenum Alloy

**DOI:** 10.3390/ma11071071

**Published:** 2018-06-25

**Authors:** Geng An, Jun Sun, Yuanjun Sun, Weicheng Cao, Qi Zhu, Qinglin Bai, Linjie Zhang

**Affiliations:** 1 State Key Laboratory of Mechanical Behavior for Materials, Xi’an Jiaotong University, Xi’an 710049, China; agmail@163.com (G.A.); junsun@mail.xjtu.edu.cn (J.S.); sunyj163@.com (Y.S.); baiqinglin@stu.xjtu.edu.cn (Q.B.); 2 Technical Center, Jinduicheng Molybdenum Co., Ltd., Xi’an 710077, China; cao_wch@163.com (W.C.); log__xy@163.com (Q.Z.)

**Keywords:** molybdenum alloy, laser welding, lap joint, fuel cladding, end plug

## Abstract

The study investigated the laser lap welding of fuel cladding and end plug made of molybdenum (Mo) alloy. The research results showed that the tensile strength of the welded joint when a weld was located at the Mo tube was significantly larger than that at the fit-up gap between the fuel cladding and end plug. Moreover, preheating can also greatly increase the tensile strength of the lap joint. The weld zone was filled with bulky coarse columnar crystal structures while there were numerous coarse recrystallized structures in the heat affected zone (HAZ). The weld zone and HAZ were both subjected to a significant softening. The tensile strength and elongation rate of fuel cladding made of Mo alloy were about 750 MPa and 36.7%, respectively. The welded joint did not undergo any plastic deformation during the tensile process and presented a brittle fracture. Under the optimum processing conditions, the tensile strength of the welded joint reached 617 MPa, taking up 82.3% that of the base metal. The results of composition analysis indicated that there was only Mo inside the columnar crystals in weld zone while significant oxygen segregation was observed at the grain boundary. This was the main reason causing that the strength of welded joint was lower than that of the base metal. Additionally, under the optimum processing conditions, there were numerous slender columnar crystals on the cross section of the joint entering the weld zone in fuel cladding side from that in end plug side where the crystals were nucleated and grew upwards. The analysis results suggested that the presence of these slender columnar crystals crossing the interface between fuel cladding and end plug was favorable for improving the capacity of the joint for bearing the shear loads.

## 1. Introduction

Since the accident of Fukushima Nuclear Power Station of Japan in 2011, the nuclear industry has realized that it is necessary to develop a new fuel system, namely, accident tolerant fuel (ATF) [1]. The fuel system needs to be able to resist serious accident conditions for a long time, delay the deterioration rate of situations, spare precious time so people can take emergency measures and greatly decrease the leaking risk of radioactive materials. Molybdenum (Mo) has many advantages including high melting point, favorable strength at high temperatures, low neutron absorption cross section, good thermal conductivity, low linear expansion coefficient, and excellent wear and corrosion resistances [2,3]. Therefore, Mo alloy is listed as one of the main selected materials for preparing ATF cladding. For this reason, Mo and Mo alloy welding technologies have been intensively concerned by scholars in recent years [4,5,6,7,8,9,10,11,12,13].

Mo and Mo alloys are essentially hard and brittle materials and therefore they generally exhibit a poor weldability and have many unsolved welding issues [14,15,16,17]. Currently, the welding methods of Mo alloy mainly involve tungsten inert gas (TIG) welding, electron beam welding (EBW), laser beam welding, electric resistance welding (ERW), brazing and friction stir welding. The strength and heat resistance of brazed welds are both lower than those of base metals and their service performance at high temperatures is basically inferior to that of the fusion-welded joint. ERW is not an effective way to weld Mo and Mo alloys owing to the high conductivity and high yield strength at high temperatures of Mo and Mo alloy. Friction stir welding of Mo and Mo alloys shows many problems such as serious wear of tools, a keyhole difficult to repair formed in welds while taking the stir-welding head from the specimen after completing the welding, decreasing corrosion resistance of welds and difficult to process thin-wall tubes. The high conductivity, significant grain coarsening and embrittlement tendencies of Mo alloy determine that it exhibits great superiorities for welding Mo and Mo alloy with a heat-source of a high-power density. The EBW is widely used in Mo and Mo alloy welding owing to it exhibits a high-power density and can prevent melted metals from being contaminated by harmful gases (such as oxygen and nitrogen) during welding in a vacuum environment. The laser beam welding with a high-power density can be used for welding refractory alloy and difficult-to-weld materials [18,19,20,21]. Moreover, the high-energy beam welding exhibits a large welding speed, a small heat affected zone (HAZ), low welding stress and small deformation [22,23,24,25,26]. Compared with the EBW, laser beam welding is more flexible and efficient because the size and shape of weldments welded using laser beam welding are not constrained by the vacuum chamber and the welding process are not affected by electromagnetic fields. Thus, many scholars have attempted to apply laser beam welding to weld the Mo alloys.

Liu et al. (2016) [27] investigated the Nd:YAG laser lap welding of powder metallurgy Mo-Re alloys (50Mo-50Re) plate with a thickness of 0.13 mm. Cracks appeared on the bonding interface of fusion zone (FZ) after welding and numerous large-size pores were observed on the lap interface. The diameter of pores was about 15–20% of the thickness of base metals. The microscopic analysis result showed that the fracture belonged to intergranular fracture and there were numerous dark compounds inside grains and on the grain boundary. The composition analysis indicated that C and O contents in the dark compounds separately take up 30 and 15 at %. Moreover, the average values of microhardnesses of base metals, HAZ and FZ after welding were 290, 370 and 420 HV, respectively, implying that the welds and HAZ after welding significantly hardened. The authors suggested that coarse structures and harmful impurity elements are the primary reason causing the hardening of the bonding interface and the intergranular fracture of lap joints. Lin (2013) [28] welded the needle-like conducting element made of pure Mo with 0.5 mm in diameter by replacing the ERW with pulsed Nd:YAG laser beam welding. The research result showed that the joint strength can be strengthened by about twice by using pulsed Nd:YAG laser beam welding.

Kramer et al. (2012) [29] investigated the EBW and the pulsed Nd:YAG laser beam welding of Mo-44.5% Re alloys sheet with 0.5 mm in thickness. The result indicated that the joint of Mo-44.5% Re alloys welded by using EBW had a favorable appearance and there were no pores and crack defects. The joint structure obtained by applying laser beam welding was tinier than that acquired using EBW. However, cracks appeared in the FZ of the Mo-44.5% Re alloy joint welded by using laser beam welding and also the fracture of the obtained joint exhibited the microstructure of brittle fracture. Chatterjee et al. (2016) [6] investigated the laser beam welding and the arc-preposition Nd:YAG laser/TIG hybrid welding for butt joint of forging Ti-Zr-Mo alloys with 1.2 mm in thickness. Pore defects did not occur in the joints welded by using the two welding methods. The FZ and HAZ of the joints welded by using the two methods separately appeared as coarse columnar crystal structures and coarse equiaxed grain structures. By contrast, the grain sizes in FZ and HAZ of joints welded by applying EBW were greatly smaller, which was separately about 55% and 65% of those of the joint obtained by using hybrid welding. The widths of welds obtained by using EBW and hybrid welding were about 1.4 and 2.6 mm, respectively, while the widths of HAZ attained using the two welding method were both 1.5 times those of the weld zone. Under the two conditions, the welds and HAZ both softened and the microhardness of FZ in the joint obtained through EBW reduced by about 26% compared with that of base metals. On the condition of using hybrid welding, there was a wider softening zone and a larger softening degree. The result of tensile test revealed that the strengths of the joints prepared by using laser-TIG hybrid welding and EBW separately accounted for about 41% and 47% of those of the base metals. Although FZ and HAZ both softened, the two joints nearly did not exhibit the tensile plasticity during the tensile test and also the fracture reduction and elongation rate were nearly equal to 0 while the elongation rate of base metals were up to 8.4%. The tensile fractures of the two joints both exhibited the brittle fracture shown as the transgranular fracture. Although EBW was operated in high vacuum environment, the TEM result for the FZ in joint welded using EBW showed that there were numerous uniformly distributed diffused second phases inside the grains with the size ranging from 0.1 to 10 μm. The composition analysis showed that these diffused second phases were the oxides of Mo, which contained about 65 at % of O and 34.5 at % of Mo. The fracture analysis and TEM test result indicated that the grain boundary segregation was the reason the elongation rate of welded joints was nearly 0.

By analyzing the current research results, it can be seen that laser beam welding exhibits many advantages such as high production efficiency, the size and shape of weldments being not restricted by vacuum chamber. However, because laser beam welding is conducted in non-vacuum environment, it is hard to acquire a high-strength joint using laser beam welding. Therefore, it is necessary to deeply explore the factors influencing the welding quality of Mo and Mo alloy through laser beam welding and the influence law to promote the application of the laser beam welding in Mo alloy welding manufacturing.

## 2. Materials and Methods

### 2.1. Experimental Materials

**Figure 1:**
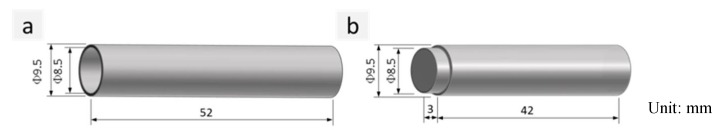
(**a**) The sizes of Mo tube; and (**b**) the sizes of Mo end plug.

La_2_O_3_ dispersion-strengthened Mo alloy was used for the test in which the content of La was about 0.25 wt % [30]. The fuel cladding and solid end plug used for the welding test were produced through a series of processes such as forging and hot rolling and their shapes and sizes are shown in Figure 1. They were grinded using sand paper before welding and then the specimen to be welded was washed by applying acetone to remove the oxide film and oil stain on the metal surface. The welding test was completed within the subsequent two hours.

### 2.2. Experimental Equipment and Method

**Figure 2:**
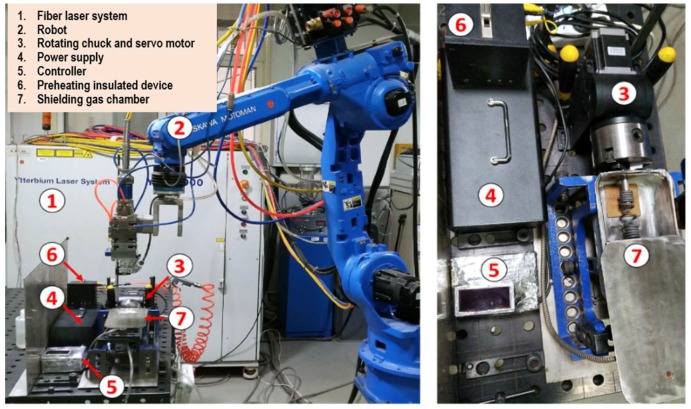
The laser beam welding system used in this work.

The laser beam welding system is composed of an IPG TLS-4000 (Apache, Oxford, OH, USA) multi-mode fiber laser, a YASKAWA HP20 robot (Yaskawa, kitakyushu, Fukuoka, Japan), rotating fixtures, a preheating insulated device and a shielding gas chamber, as shown in Figure 2. The maximum output power, the diameter of transmission fiber, the diameter of the focusing lens, the focal length and the minimum spot diameter of the laser system were 4 kW, 0.2 mm, 50 mm, 150 mm and 0.2 mm, respectively. The laser head was installed on the arm end of YASKAWA HP20 robot. The rotation speed of the rotating chuck ranged from 0.1 to 60 r/s. During welding, the shielding gas chamber was filled with high-purity argon (i.e., 99.9999%) to prevent that the high-temperature area of the specimen from contamination.

The cross-sections of the welded joints were ground, polished, etched and observed by using the Nikon Eclipse MA200 (Minato-ku, Tokyo, Japan) optical microscope and the LS-JLLH-22 scanning electron microscope (SEM) ( HITACHI, Tokyo, Japan). The etchant consisted of 10 mL hydrogen fluoride (HF), 10 mL HNO_3_ and 80 mL water. The Vickers microhardness test was performed on the cross-sections of the welded joints using a 200 g load for 15 s to obtain the hardness profiles. The tensile test was conducted on a universal mechanical testing machine with a drawing speed of 1.0 mm/min. The microhardness distribution of the cross section of the welded joint was measured under the loading load of 200 gf and the holding time of 15 s. The tensile test of the lap joint was conducted on the Instron universal mechanical testing machine with the tensile speed of 1.0 mm/min. Afterwards, a LS-JLLH-22 type scanning electron microscope (SEM) was used to observe the tensile fractures and energy spectrum analysis (EDS) was employed to detect the element composition of the fracture.

## 3. Experimental Results and Discussion

### 3.1. Effects of Weld Position and Preheating

**Figure 3:**
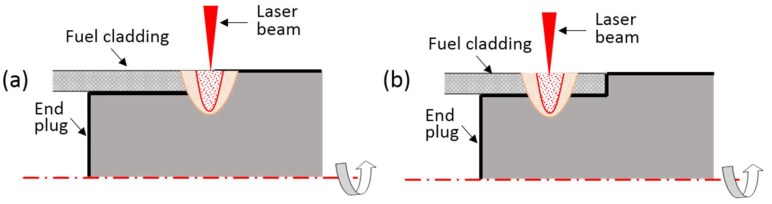
The weld positions: (**a**) at the shoulder of end plug; and (**b**) at tube.

**Figure 4:**
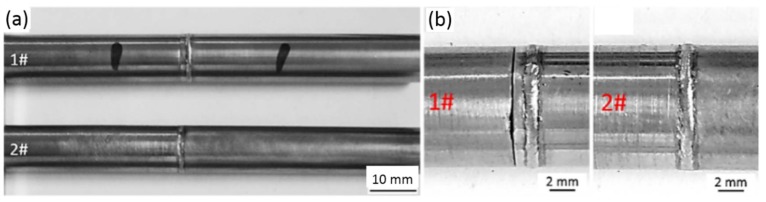
The surface morphology of welded joint with different weld positions: (**a**) Whole welded specimen; and (**b**) welded joint.

At the beginning, the influences of weld position and preheating on the joint strength were explored. Figure 3 shows the two kinds of weld position considered in the work. Figure 4 shows the microstructures of welded specimens at two weld positions. Table 1 lists the tensile strengths of welded joints produced under three different conditions.

By comparing the tensile strengths of Case No. 1 and Case No. 2 in Table 1, it can be found that the joint of which the weld position was at the tube side has higher strength than the joint of which the weld position was at the shoulder of end plug. Additionally, the results showed that tensile strength of joints could be significantly improved through preheating, as indicated by the results of Case No. 1 and Case No. 3 in Table 1.

### 3.2. Optimization of Welding Parameters

**Figure 5:**
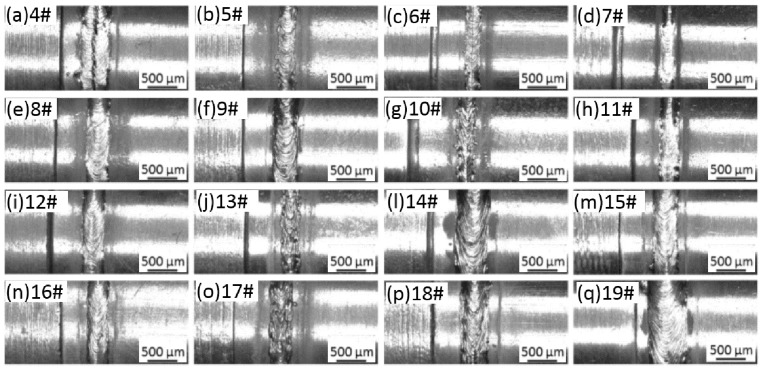
A comparison of weld morphologies based on the three-factor and four-level orthogonal test: (**a**–**q**) orthogonal parameter table 2 number 4–19 specimens.

Afterwards, the study formulated the three-factor and four-level orthogonal test scheme by taking the laser power P, the defocusing amount f and the welding speed v as variables to optimize the welding parameters of laser beam welding. Table 2 displays the test scheme and test results of tensile strength of the corresponding joints. Figure 5 shows the weld microstructures obtained through welding with parameters in Table 2. In the test, the weld position was at the Mo tube side, as shown in Figure 3b. The specimen was preheated to about 450 °C before the welding. The range analysis was conducted on the results of the tensile strength and the analysis results showed that the welding speed (v) exhibited the most significant influence on the tensile strength of the joint, followed by the laser power (P), and the defocusing amount (f) exerted the lowest influence.

**Figure 6:**
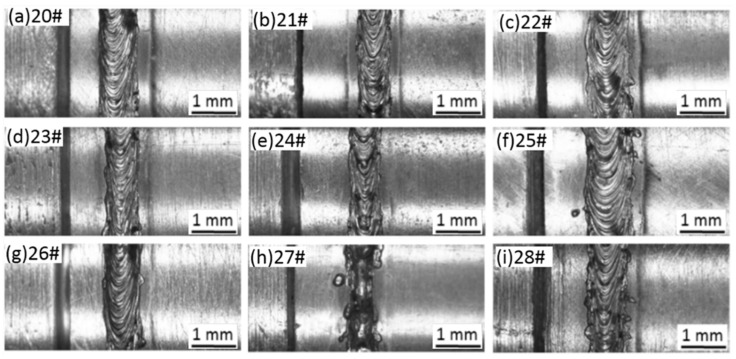
A comparison of weld morphologies obtained by using three-factor and three-level orthogonal test scheme: (**a**–**j**) orthogonal parameter table 3 number 20–28 specimens.

Through the analysis of the results of range and variance, the influence of different factors (i.e., laser power, defocus amount and welding speed) on the tensile strength of laser welded joint can be determined to judge whether the influence of each factors on the test index (i.e., tensile strength) is significant, thereby optimizing the process parameters. We have found that when the power is around 2500–3000 W, the defocus amount is 1 mm, and the welding speed is around 5 m/min, a higher tensile strength can be obtained. The study further optimized the welding technological conditions by formulating the three-factor and three-level orthogonal test scheme, as listed in Table 3. According to Table 2, the welded joints prepared under the three technological conditions (i.e., Numbers 13, 14 and 16) exhibited a high tensile strength. Among these three conditions, although the welded joint achieved under the technological condition of Number 13 showed a lower strength, its weld width was significantly narrower than those of the other two welded joints. Considering the high thermal conductivity of Mo alloy and significant coarsening and embrittlement tendencies of the welded joint of Mo alloy, it is speculated that better results might be achieved under an optimal combination of technological parameters around the condition of Number 13 (i.e., P = 2500 W, f = 0 mm and v = 5 m/min). Therefore, the study further formulated a three-factor and three-level orthogonal test scheme, as shown in Table 3. The corresponding test results of tensile strengths are also displayed in Table 3. It can be seen in the table that the tensile strength of welded joints through further optimization reached about 617.18 MPa under the condition of P = 2500 W, f = 1 mm and v = 6 m/min. Figure 6 shows the surface morphology of welds produced based on the parameters in Table 3. 

### 3.3. Microstructure and Mechanical Properties

**Figure 7:**
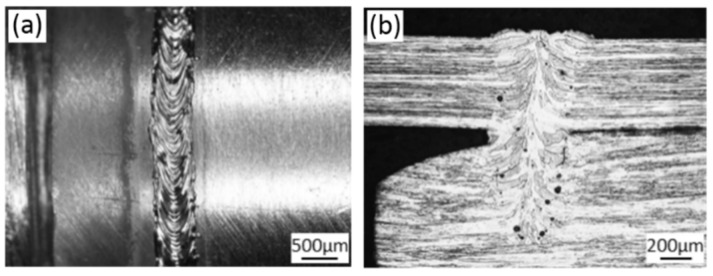
The surface and cross-section morphologies of the lap joint obtained through laser beam welding (laser power P = 2500 W, defocusing amount f = 1 mm, welding speed v = 6 m/min and preheating at 450 °C): (**a**) welded joint surface morphology; and (**b**) welded joint cross-section morphology.

Figure 7a,b separately displays the surface morphology and cross-section of the laser welded joints with a tensile strength of 617.18 MPa. It can be seen in Figure 7a that the weld surface showed bright metal luster, which implies that the heated area was favorably protected during welding. As shown in Figure 7b, the longitudinal sections of base metals of fuel cladding and end plug made of Mo alloy both appeared as typical rolled microstructure in which the lamellar microstructure of the base metal of fuel cladding was tinier. Mo and Mo alloy exhibited unique characteristics including high melting point, good thermal conductivity, high recrystallization temperature and having no allotropic transformation under a solid state, and the less compact bcc crystal of Mo is in favor of diffusion. Therefore, the grain coarsening tendency was serious at the weld and HAZ of Mo alloy joint. It can be seen in Figure 7b that the weld zone was filled with coarse columnar crystals while the HAZ near the weld zone exhibited coarse recrystallized microstructure. The grain length of columnar crystals in the weld zone was about half of the weld width. The columnar grains first grew to the weld center from the FZs, then upward deflected near the weld center and finally converged at the weld center.

Additionally, as displayed in Figure 7b, pores with the diameter of 10–50 μm appeared near the fusion line. At the late period of sintering of powder metallurgy, although the sintering lasted for a long time, some residual small pores were not removed but become residual pores, causing the formation of pore defects during welding. Xu et al. (2012) found that the FZs of lap joints of the powder metallurgy 50Mo-50Re (wt %) produced through resistance spot welding under various welding conditions all exhibited large-size pore defects [9]. Liu et al. (2016) suggested that there were numerous large-size pores near the interface of the laser welded lap joint of powder metallurgy Mo-Re alloys (50Mo-50Re) with the thickness of 0.13 mm [27]. Stütz et al. (2016) observed that the pore defect was more serious when the heat input was large during EBW welding of TZM alloy with a thickness of 2 mm [4]. This did not conform to the common knowledge: the welding under a large heat input is favorable for the bubble emersion in molten pool and therefore effectively inhibits the pore defect. M. Wahba et al. investigated the formation mechanism of pore defect during laser welding of powder metallurgy AZ31B Mg alloy [31]. By conducting the X-ray transmission imaging, they found that the bubbles in molten pool were not caused due to unstable keyholes but released from the base metal.

**Figure 8:**
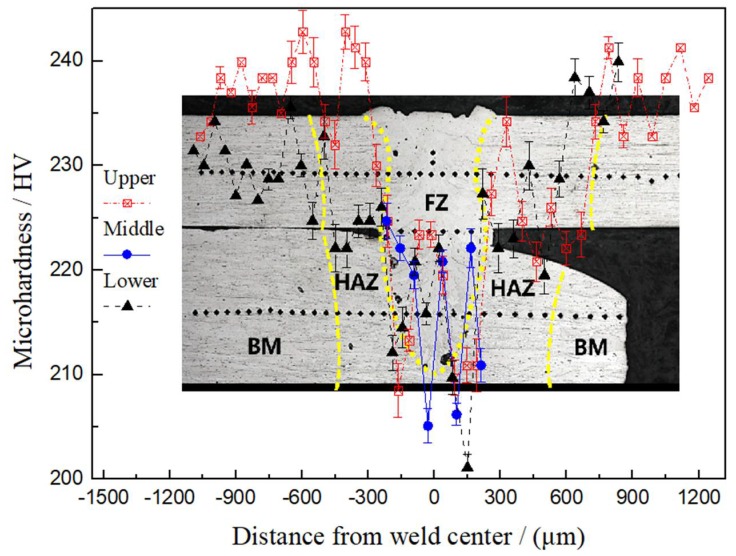
Microhardness distribution of the cross section of the joint using laser beam welding (laser power P = 2500 W, defocusing amount f = 1 mm, welding speed v = 6 m/min and preheating at 450 °C).

Figure 8 displays the microhardness distribution on the cross section of the welded joint with a tensile strength of 617.18 MPa. As shown in the figure, the microhardnesses of the base metals of Mo tube and rod were about 240 and 230 HV, respectively. The weld zone and HAZ both exhibited a significant softening phenomenon. The weld zone had the lowest microhardness (i.e., 200–220 HV) while the microhardness of the HAZ gradually transmitted to that of the base metal from the value near the fusion line (i.e., about 220 HV).

**Figure 9:**
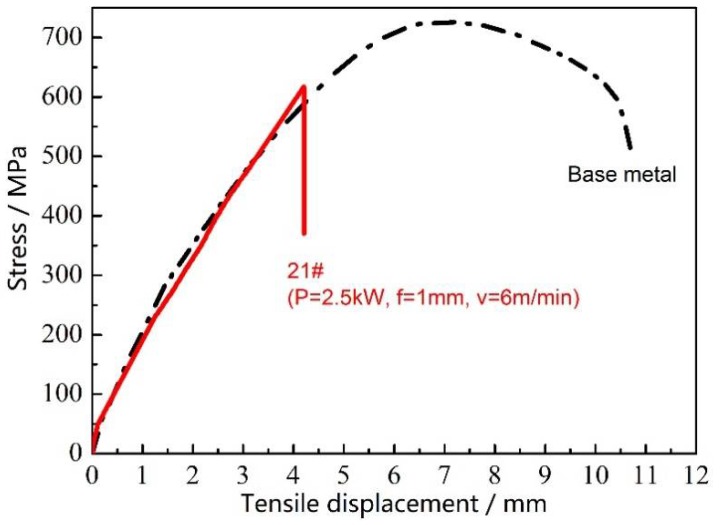
The tensile test result of the base metal of Mo tube and the joint welded through laser beam welding (laser power P = 2500 W, defocusing amount f = 1 mm, welding speed v = 6 m/min and preheating at 450 °C).

Figure 9 compares the tensile test results of the welded joints achieved under optimized welding conditions and that of the base metals of Mo tube. As shown in the figure, the tensile strength of the base metal of Mo tube reached about 720 MPa. In the tensile process, the base metal of Mo tube underwent a great plastic deformation and the elongation rate was about 36.7%. On the other hand, the tensile strength of the welded joint by using optimized welding condition reached about 617 MPa, which was about 82.3% that of the base metal. The toughness of Mo and Mo alloy varied with temperatures and the fracture of Mo alloy transformed from ductile into brittle fracture within a narrow temperature range. The pure Mo was subjected to the ductile–brittle transformation at 140–150 °C, which was attributed to the intrinsic brittleness of Mo. Although the base metal of Mo tube itself exhibited favorable strength and toughness after undergoing the nano-sized doping strengthening and toughening treatment, the strengthening and toughening effects of the base metal would be lost once melting and solidification appeared during welding. It can be seen in Figure 9 that the welded joint was basically not subjected to plastic deformation during the whole tensile process but shown as the brittle fracture. 

### 3.4. Grain Boundary Segregation

**Figure 10:**
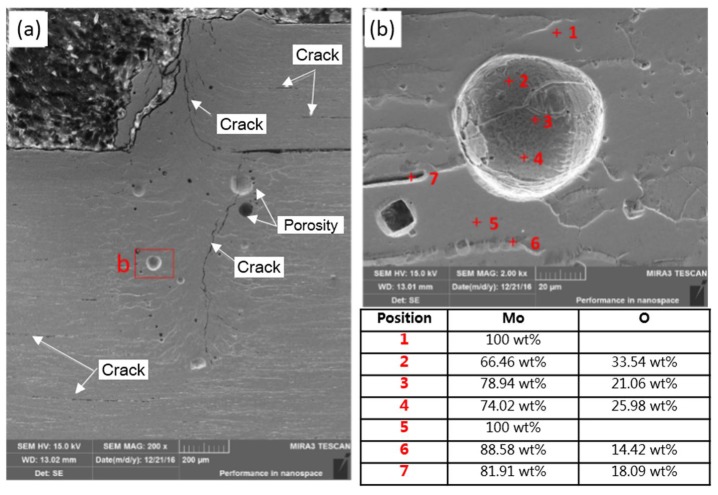
The cross-section morphology and component analysis of the welded joint prepared using laser beam welding after the tensile fracture (laser power P = 2500 W, defocusing amount f = 1 mm, welding speed v = 6 m/min and preheating at 450 °C): (**a**) cross-section morphology; and (**b**) component analysis of the welded joint.

Figure 10 displays the cross-section morphologies of the welded joint prepared by using optimized welding technology after the tensile fracture. As shown in Figure 10a, numerous small pores with the size about 10–20 μm and few macropores with the size of 100–200 μm were distributed in the weld. Therein, large-size pores mainly appeared near the fusion line while small-size pores both occurred near the fusion line and the middle part of the weld. It can be clearly seen in Figure 10a that the majority of small-size pores distributed along the fusion line appeared as chainlike distribution. Although there were numerous pores in the weld zone, the cracking position did not occur in priority at the pores (Figure 10a). According to the literature, this was because the coarse grain boundary in the weld zone and grain boundary segregation of oxides of Mo resulted in the significant reduction of grain boundary strength.

**Figure 11:**
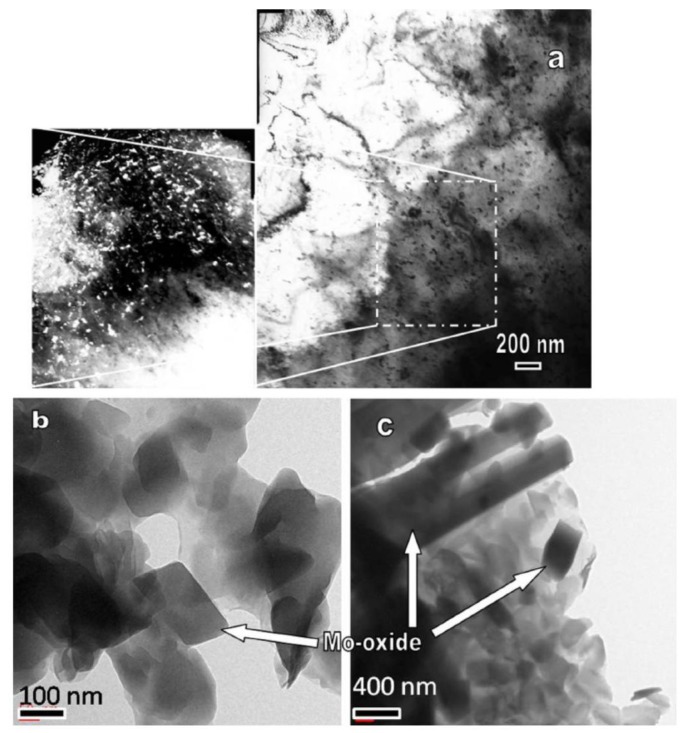
TEM observation of the FZ of EBW welded TZM alloy [6]: (**a**) the presence of the precipitates within the matrix. Inset shows the magnified view; (**b**) Magnified view showing the presence of Mo-oxide phase in the weld region; and (**c**) The presence of needle-shaped long oxides may be noticed.

It can be seen from the component analysis result of Figure 10b that the internal part of the columnar crystals in the weld zone was pure Mo while there was significant O segregation at the grain boundary. It can be seen in Figure 10b that the internal wall of pores exhibited a high O content to 33.54 wt %. It was likely to have MoO_3_ on the internal wall of pores. Owing to the solid solubility of O in Mo being low, the inner of grains appeared as pure Mo and the O enrichment occurred at the grain boundary while the grains in the weld zone were greatly coarsened and the grain boundary area significantly reduced. Therefore, O element was greatly segregated at the grain boundary. Numerous researches have reported that the segregation of O element at the grain boundary of Mo caused the production of volatile Mo oxide at the grain boundary to further reduce the strength of grain boundary and significantly decrease the toughness of the joint. Chatterjee et al. (2016) observed the O segregation at the grain boundary from the FZ of the welded joint of Ti-Zr-Mo alloys (Ti 0.50 wt %, Zr 0.08 wt % and C 0.04 wt %) through EBW [6]. They also found numerous uniformly distributed second phases in crystals in the FZ. Their component analysis revealed that these second phases were the oxides of Mo, which contained 65 at % of O and 34.5 at % of Mo, as shown in Figure 11. Owing to the welding being conducted in a vacuum chamber, Chatterjee suggested that O in the base metal was redistributed during the welding to cause the production of diffused second phases in grains and O segregation at the grain boundary. In this way, the strength of the grain boundary significantly reduced. It can be observed in Figure 10a that there were numerous cracks initiating along the grain boundary of columnar crystals on the cross section of the welded joint after the tensile fracture. Additionally, some cracks parallel to the tensile direction (horizontal direction) also occurred in the base metals of Mo tube and the end plug, which was mainly related to the lamellar structure produced in the rolling process of base metals. It should be noticed here that the oxygen content given in Section 3.4 was determined by EDS, and the results of oxygen content were qualitative rather than quantitative.

### 3.5. Fracture Observation

**Figure 12:**
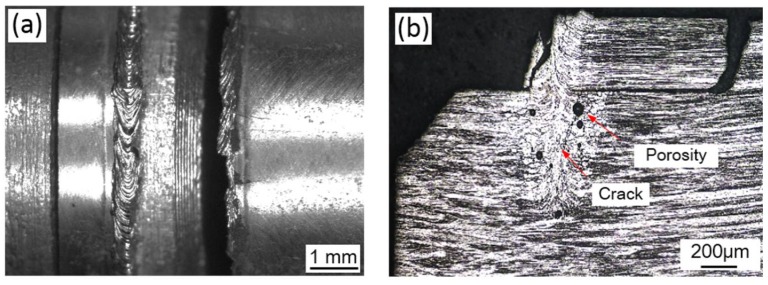
The surface and cross-section morphologies of the joint welded using laser beam welding after the tensile fracture (laser power P = 2500 W, defocusing amount f = 1 mm, welding speed v = 6 m/min and preheating at 450 °C): (**a**) welded joint surface morphology; and (**b**) welded joint cross-section morphology.

The surface morphology of the welded joint and the cross section of the weld after the tensile fracture are shown in Figure 12. As displayed in Figure 12a, part of the tensile fracture was located in the weld center while the other part appeared near the fusion line. Figure 12b shows that, near the lap interface of the Mo tube and end plug made of Mo, the slender columnar crystals nucleated from the side of the end plug grew upwards along the vertical direction to the weld zone in the side of the Mo tube. The slender columnar crystals crossing the fuel cladding/end plug interface were subjected to a significant plastic deformation along the shear direction. It was speculated that the favorable tensile strength of the joint was attributed to the slender columnar crystals crossing the fuel cladding/end plug interface. The presence of the structure was in favor of improving the capacity of welded metals at the interface of fuel cladding/end plug for bearing the shear loads. 

**Figure 13:**
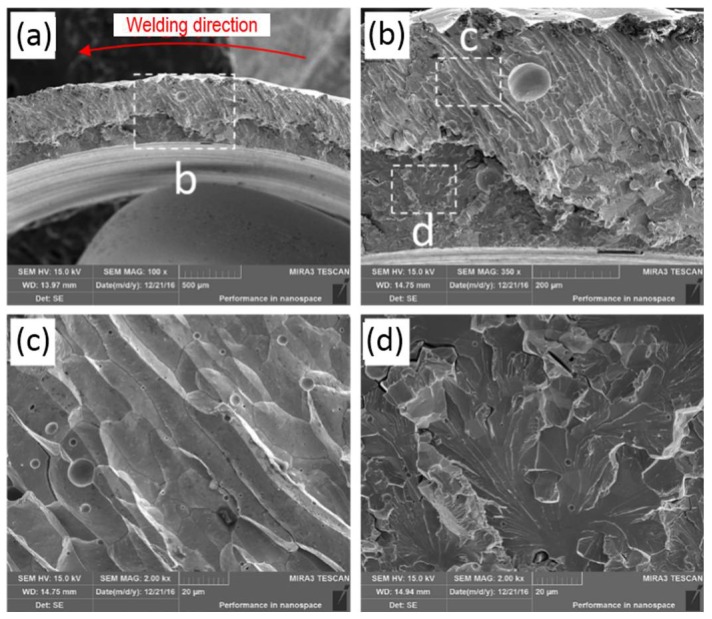
The SEM morphologies of the tensile fracture of the joint welded using laser beam welding (laser power P = 2500 W, defocusing amount f = 1 mm, welding speed v = 6 m/min and preheating at 450 °C): (**a**) fracture morphology; (**b**) an enlarged view of area b in (**a**); (**c**) an enlarged view of area c in (**b**); and (**d**) an enlarged view of area d in (**b**).

**Figure 14:**
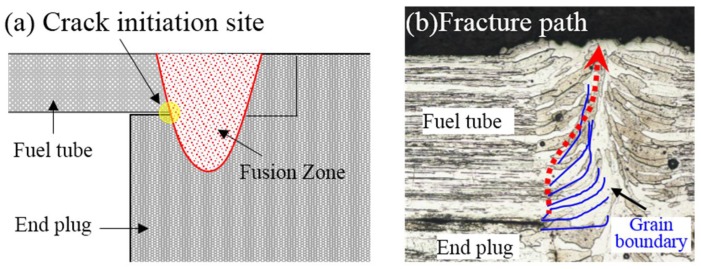
The formation mechanism of intergranular and transgranular fractures on the tensile fracture of the joint welded applying laser beam welding: (**a**) crack initiation site schematic; and (**b**) fracture path schematic.

Figure 13 displays the SEM observation result of the tensile fracture of the welded joint prepared using laser beam welding. It can be seen in Figure 13a that the fracture morphology near the external surface of the Mo tube was very different from that near the internal surface. As displayed in Figure 13b–d, the fracture near the external surface of Mo tube was the intergranular cracking, shown as fluctuated slender rod-like morphology, while the columnar grains approximately grew along the radial direction of the Mo tube. By contrast, the fracture near the internal surface of the Mo tube belonged to transgranular cracking. By observing the partially enlarged picture in Figure 13c, it can be seen that the rod-like morphologies were actually coarse columnar crystals with flat and smooth surfaces and different sizes of pores were distributed near the columnar grain boundary. As shown in the partially enlarged picture in Figure 13d, the fracture near the internal surface of the Mo tube was flat and there were numerous river-like patterns on the fracture, which appeared as typical transgranular cleavage fracture. As displayed in Figure 14, the columnar crystals in the weld zone grew along the horizontal direction near the fusion line at first and then gradually rose upwards along the vertical direction near the weld center. During the tensile test, the stress concentration phenomenon occurred at the intersection point of the fusion line and the interface of fuel cladding/end plug, which caused the crack initiation. The initiating cracks first crossed the columnar crystals near the fusion line growing along the horizontal direction, grew upwards to a certain distance along the vertical direction and then propagated along the boundary of columnar grains. Finally, the welded joint was completely fractured to form the fracture morphology shown in Figure 13a,b.

## 4. Conclusions

The study investigated fiber laser beam welding for the lap joint of fuel cladding and end plug made of La_2_O_3_ dispersion-strengthened Mo alloy. The main conclusions are displayed as follows:

(1) Changing the seam position from the joint of fuel cladding and end plug to the Mo tube can significantly improve the tensile strength of the joint. Preheating before welding can also greatly enhance the tensile strength of the lap joint.
(2) The longitudinal cross sections of the base metals of the fuel cladding and the end plug made of Mo alloy were typical rolling structure while the HAZ appeared as the recrystallized structure. The weld zone was full of coarse columnar structures. Moreover, the weld and HAZ significantly softened after the welding.
(3) The inner of the columnar crystals in the weld zone was filled with 100 wt % Mo while 14–18 wt % of O content was found at the grain boundary in the weld zone. The O content on the internal walls of pores reached up to 33 wt %.
(4) The tensile strength and elongation rate of fuel cladding made of Mo alloy were 750 MPa and 36.7% while the tensile strength of the welded joint was 617 MPa, which was about 82.3% of the base metal. The welded joint was basically not subjected to the plastic deformation during the whole tensile process but showed brittle fracture.
(5) There were numerous slender columnar crystals on the cross section of the joint entering the weld zone of fuel cladding from that of end plug where the crystals were nucleated and grew upwards. The presence of these slender columnar crystals crossing the interface of fuel cladding and end plug was favorable for enhancing the capacity of the joint for bearing the shear loads.

Finally, considering that millions of human lives are involved, the authors have to point out that the current work is only a preliminary study on the weldability of fuel cladding and end plug made of La_2_O_3_ dispersion-strengthened molybdenum alloy and there is still a long way to go before applying it in the nuclear industry.

## Figures and Tables

**Table 1:** Influences of weld position and preheating on joint strength.

Number	Weld Position	Laser Power (W)	Defocusing Amount (mm)	Welding Speed (m/min)	Preheating Temperature (°C)	Tensile Strength (MPa)
1	Tube	2000	2	2	450	280.1
2	Shoulder of end plug	2000	2	2	450	126.8
3	Tube	2000	2	2	-	66.3

**Table 2:** Three-factor and four-level orthogonal test of continuous laser beam welding.

Number	Laser Power P (W)	Defocusing Amount f (mm)	Welding Speed v (m/min)	Cracks	Weld Position	Preheating Temperature (°C)	Tensile Strength (MPa)
4	1500	−2	2	Yes	Tube	450	0
5	1500	0	3	No	Tube	450	252.12
6	1500	2	4	Yes	Tube	450	99.19
7	1500	4	5	No	Tube	450	52.79
8	2000	−2	3	No	Tube	450	229.87
9	2000	0	2	No	Tube	450	213.00
10	2000	2	5	No	Tube	450	219.00
11	2000	4	4	Yes	Tube	450	29.00
12	2500	−2	4	Yes	Tube	450	98.00
13	2500	0	5	No	Tube	450	426.58
14	2500	2	2	No	Tube	450	436.00
15	2500	4	3	Yes	Tube	450	163.70
16	3000	−2	5	No	Tube	450	517.81
17	3000	0	4	No	Tube	450	209.98
18	3000	2	3	No	Tube	450	141.14
19	3000	4	2	No	Tube	450	165.00

**Table 3:** Three-factor and three-level orthogonal test of continuous laser beam welding.

Number	Laser Power P (W)	Defocusing Amount f (mm)	Welding Speed v (m/min)	Tensile Strength (MPa)
20	2500	0	4	285.08
21	2500	1	6	617.18
22	2500	2	5	373.98
23	2800	0	6	505.17
24	2800	1	5	253.82
25	2800	2	4	365.26
26	3000	0	5	376.10
27	3000	1	4	369.62
28	3000	2	6	388.02
